# Interactions between gut microbes and host promote degradation of various fiber components in Meishan pigs

**DOI:** 10.1128/msystems.01500-24

**Published:** 2025-01-28

**Authors:** Guang Pu, Liming Hou, Qingbo Zhao, Gensheng Liu, Zhongyu Wang, Wuduo Zhou, Peipei Niu, Chengwu Wu, Pinghua Li, Ruihua Huang

**Affiliations:** 1 Key Laboratory of Pig Genetic Resources Evaluation and Utilization (Nanjing), Ministry of Agriculture and Rural Affairs, Institute of Swine Science, College of Animal Science and Technology, Nanjing Agricultural University 70578 , Nanjing, China; 2 Laboratory of Intestinal Microbiology, Huaian Academy, Nanjing Agricultural University, Nanjing, China; 3 Industrial Technology System Integration Innovation Center of Jiangsu Modern Agriculture (PIG), Nanjing, China; California State University Stanislaus, Turlock, California, USA

**Keywords:** Meishan pigs, dietary fiber, fiber-degrading microbes and SCFA transport-related candidate gene

## Abstract

Although metagenomic investigations into microbial fiber-degrading capabilities are currently prevalent, there is a notable gap in research concerning the regulatory mechanisms underpinning host-microbiota interactions that confer tolerance to high-fiber diets in pigs. In this study, 28 Meishan (MS) and 28 Large White (LW) pigs were subjected to feeding experiments involving various fiber levels. Subsequently, multi-omics was employed to investigate the influence of host-microbiota interactions on the fiber degradation of pigs. MS exhibited superior fiber digestibility compared with LW, particularly evident when fed a high-fiber diet. In MS, positive interactions among *Treponema bryantii*, *Treponema* sp., *Rikenellaceae* bacterium, and *Bacteroidales* bacterium WCE2004 facilitated the degradation of both cellulose and pectin. The reduced polymerization of polysaccharides and oligosaccharides observed in MS provides compelling evidence for their superior microbial fiber-degrading capability. The concentrations of propionate and butyrate retained in cecal lumen of MS was unchanged, whereas it was significantly increased in LW, indicating a strong absorption of short-chain fatty acids (SCFAs) in MS intestines. Correlation analysis using RNA-seq data revealed distinct patterns in LW and MS. In LW, microbial profiles along with *GPR183* and *GPR174* exhibited negative correlations with butyrate and propionate, respectively. Conversely, in MS, *GPR174* and *SLC2A4* were positively correlated with butyrate. Our findings underscore the dynamic collaboration among microbial species in degrading cellulose and pectin, coupled with the synergistic effects of SCFA transport-related genes, as crucial underpinnings for the heightened fiber digestibility observed in MS. These discoveries offer fresh perspectives into the intricate mechanisms governing host-microbiota interactions that influence fiber digestion in pigs.

**IMPORTANCE:**

Studies on porcine intestinal microbiota have been widely conducted, and some microbial taxa with fiber degradation functions have been identified. However, the mechanisms of division among gut microbes in the degradation of complex fiber components are still unclear. In addition, the regulation of fiber digestion by host through absorption of short-chain fatty acids (SCFAs) needs to be further investigated. Our study used apparent total tract digestibility of dietary fiber to assess the utilization efficiency of dietary fiber between Meishan and Large White pigs. Subsequently, through metagenome sequencing and determination of fiber-degrading products, we found that in Meishan pigs, positive interactions among *Treponema bryantii*, *Treponema* sp*.*, *Rikenellaceae* bacterium, and *Bacteroidales* bacterium WCE2004 facilitated the degradation of both cellulose and pectin. RNA-seq analysis elucidated breed-specific genes associated with SCFA absorption in cecum. By integrating multi-omics data, we constructed a framework outlining host-microbiota interactions that control dietary fiber utilization in pigs. Our data provide novel insights into host-microbiota interactions regulating fiber degradation and lay some theoretical foundations for improving the utilization efficiency of high-fiber cereal feed in pigs through targeted modulation of gut microbial function.

## INTRODUCTION

Swine is an important source of animal protein for human consumption (1). To meet the growing demand for pork, the scale of pig breeding mainly based on corn-soybean meal diets is expanding, which leads to increasingly serious food competition between humans and livestock (2, 3). To mitigate this phenomenon, people started to develop the use of grain feeds characterized by their high-fiber content and substantial levels of anti-nutritional factors, given their global productivity and abundance of protein and energy (4, 5). It is well known that the digestion of dietary fiber in pigs depends mainly on the fermentation of their hindgut microbiota (6), and it has been shown that the ability of pigs to utilize dietary fiber is breed-heterogeneity. Urriola et al. (7) found that Meishan pigs (a well-known Chinese local pig breed) had a greater apparent total tract digestibility (ATTD) of total dietary fiber than Yorkshire pigs. Fevrier et al. (8) used a basal diet and a high-fiber diet with 51.8% wheat bran replacing a basal diet to feed Meishan and Large White pigs separately and found that ATTD of all fiber materials (crude fiber, neutral detergent fiber [NDF], and acidic detergent fiber [ADF] was significantly higher in Meishan pigs than in Large White pigs in high-fiber diets. In addition, recent studies identified that significant differences in compositions and functions of gut microbiota of different pig breeds (9–11). Based on these studies, we hypothesized that breed heterogeneity of the intestinal microbiota might influence the efficiency of dietary fiber utilization in pigs.

*In vitro*, the ability of microbes to degrade fiber is usually evaluated indirectly by measuring short-chain fatty acid (SCFA) production and pH of the culture. (12, 13), whereas methods for assessing the extent of fiber degradation in the gastrointestinal tract are still not straightforward. The degradation of dietary fiber begins with degradation of cellulose macromolecules into smaller ones, which are then sequentially degraded into oligosaccharides, disaccharides, and monosaccharides (14). Thus, during the limited fermentation time in pig hindgut, dietary fiber will be degraded by microbiota into carbohydrate molecules in different polymeric states (macromolecular cellulose, polysaccharides, oligosaccharides, and monosaccharides). Reasonably, the distribution of these carbohydrates with different degrees of aggregation in pig hindgut mirrors the dynamic progress of microbial fermentation of fibers. Therefore, unlocking the dynamic distribution of fiber substances in pig hindgut using chromatographic technologies is currently the first time and could provide a sufficient theoretical basis for investigating fiber-degrading ability of microbiota *in vivo*.

SCFAs, being the end products of microbial fermentation of fibers, are swiftly absorbed by the pig hindgut upon production to engage in the host’s metabolism (15). The anion exchange mechanism is the main mode of SCFA uptake by intestinal epithelial cells. Studies have identified various SCFA transport, including SLC5A, SLC16A, SLC26A, and SLC22A families (16, 17). Wang et al. found that the addition of dietary fiber caused changes in the concentration of SCFAs in the intestinal lumen of pigs and that addition of fiber caused an increasing mRNA expression of sodium-coupled monocarboxylate transporter 1 (*SMCT1*) and monocarboxylate transporter 1 (*MCT1*) (18). Xue et al. discovered the key individual microbial genomes and epithelial cell subtypes involved in fiber digestion, SCFA uptake, and metabolism, respectively, in the rumen (19). Based on these findings, we hypothesized that rapid transport and metabolism of SCFAs by intestinal epithelium ensures continued absorption of SCFAs in the intestinal lumen, which might collaboratively facilitate the microbial degradation of dietary fiber. The differences in fiber utilization efficiency among pigs may be related to breed-heterogeneity expression characteristics of SCFA absorption and metabolism-related genes.

Our study comprehensively assessed the dietary fiber utilization efficiency between Meishan and Large White pigs, utilizing fiber ATTD as a benchmark. Employing metagenome sequencing, SCFA analysis, and fiber-degrading substance assays, we delved into breed-specific microbial signatures and their fiber-degrading capabilities. Additionally, RNA-seq analysis illuminated breed-specific genes implicated in SCFA absorption within cecum. By integrating multi-omics data, we constructed a framework outlining breed-specific microbiota-host interactions that govern dietary fiber utilization in pigs. This comprehensive study offers invaluable insights into host-microbiota interplay regulating fiber utilization, establishing a theoretical basis for improving high-fiber grain feed efficiency in pigs via targeted intestinal microbial function modulation.

## MATERIALS AND METHODS

### Animals and experimental design

A total of 28 Meishan (MS) barrows (67.08 ± 1.53 kg initial body weight [BW]) and 28 Large White (LW) barrows (81.04 ± 1.64 kg initial BW) at the same physiological stage (20) as Meishan were used in this study (at the middle stage of fattening for both MS and LW). In each breed, pigs were distributed using a completely randomized experimental design within one of four treatments: (i) basal diet (CON), (ii) wheat bran (WB)-7, 7% wheat bran replaced basal diet, (iii) WB-10.5, 10.5% wheat bran replaced basal diet, and (iv) WB-14, 14% wheat bran replaced basal diet. A total of eight pens (2.5 m width × 5.25 m length) were used to house all pigs with seven pigs in each pen. All pens were equipped with the Osborne Testing Stations Systems (OTSS, provided by OSB Livestock Technology Co., Ltd. Shanghai, China), which can accurately record daily intake and body weight individually. Each pig is identified as a replicate, and hence, seven replicates were noted to be in each pen. The whole process consisted of a 7-day pre-feeding period followed by a 28-day experimental period. All pigs had free access to water via a low-pressure bowl drinker. The temperature and relative humidity of the pig house were maintained at 23.55°C ± 2.14°C and 69.51% ± 2.03% using direct-current frequency conversion floor heating air conditioner (Guangdong Eelaix Environmental Technology Co., Ltd. Guangdong, China). Due to the National Research Council (NRC) did not have a specific nutritional standard for LW (NRC is only applicable to Duroc × Landrace × Yorkshire) (21), therefore, we provided two pig breeds in the control with the same basal diet according to the Nutrient Requirements of Swine (GB/T 39235–2020), which has a lower nutritional level, especially for LW. The lower nutritional standard might stimulate the degradation of dietary fiber components by intestinal microbiota to compensate for the energy required by the host (6), which would be beneficial to our study of the fiber-degrading function of intestinal microbiota. The diets were produced by Huaian AnYou Feed Co., Ltd. (Jiangsu, China). To keep the nutritional level of the four diets consistent after using different levels of wheat bran to replace the basal diet, the proportion of soybean meal, soybean oil, amino acids, and other substances in the diet were slightly adjusted (Table S1). All pigs were healthy and did not receive any antibiotics during the whole experimental period.

### Sample collection

Upon completion of the experiment, prior to slaughter, fresh feces from each pig were collected. In order to avoid a decrease in the accuracy of ATTD measurement caused by fresh fecal samples falling on the ground and being contaminated with ash, the collection process needs to be strictly required: When the pig was defecating or was about to defecate, one of person quickly caught the feces with a self-zip plastic bag so that the feces do not touch the ground. Meanwhile, another person labeled the pig with spray lacquer. Through the above fecal collection methods, we can trace the fecal samples and the pig’s ID clearly and ensure that the fecal sample of each pig is in order. Then feces samples were preserved at −20°C for subsequent determination of ATTD of dietary fiber and observation of the microscopic morphology of dietary fiber remnants in the feces. Following slaughter, fresh cecal contents and cecal tissue were promptly harvested and immediately shock-frozen in liquid nitrogen to preserve their integrity. These samples were then stored at −80°C, preparing them for shotgun metagenome sequencing, quantification of fiber degradation products, measurement of short-chain fatty acids (SCFAs), and tissue RNA extraction.

### Growth performance, ATTD of dietary fiber

Based on the real-time data of daily intake and body weight of each pig recorded by OTSS, the average daily feed intake (ADFI), average daily gain (ADG), and feed-to-gain ratio (F/G) were calculated.

Naturally occurring acid-insoluble ash (AIA) of diets and feces was used as an endogenous marker for calculating ATTD of dietary components in this study (22). About 200 g of feces or diets per sample were fully dried (100°C for 4 h). AIA of diets and ingredients were firstly analyzed (Method 942.05 [23]), insoluble dietary fiber (IDF), soluble dietary fiber (SDF), and total dietary fiber (TDF) in diets and ingredients were determined with the Total Dietary Fibre Assay kit (Megazyme, Bray, Ireland), following the manufacturer’s instructions (AOAC 991.43) (23). There were three technical replicates per sample.

The ATTD of all nutrients was calculated as follows:

CAD_D_ (％) = 100 × [1 − (DC_F_ × AIA_D_) / (DC_D_ × AIA_F_)]

where CAD_D_ is the ATTD of nutrients in experimental diets, DC_F_ is the nutrient content in feces, AIA_D_ is the acid-insoluble ash in feeds, DC_D_ is the nutrient content in feeds, and AIA_F_ is the acid-insoluble ash content in feces.

### Microstructure of dietary fiber in feces

Scanning electron microscopy (SEM) is a reliable method for observing the microstructure of plant cells (24). Jia et al*.* performed SEM to observe the microstructure of SDF from defatted rice bran before and after *Trichoderma viride* fermentation (25). Along the lines of that study, SEM was used to investigate the fiber microstructure in feces fermented by cecal microbiota in this study. To remove moisture from the sample and prepare for SEM by keeping the sample in an insulated state, the fecal sample was slowly dried at 60°C (avoiding damage to the microstructure of the sample caused by high temperature). After all samples were insulated, the Vacuum ion sputter coater (Q150R ES, Judges Scientific plc, London, UK) sprayed a layer of gold film on the surface of the dry sample. Then, the samples with a layer of gold film were observed using a scanning electron microscope (Quanta FEG250, FEI company, Oregon, USA).

Plant cell wall is mainly composed of the ordered arrangement of cellulose microfibrils, which are coated with a layer of matrix polysaccharide (26). In this current study, SEM analysis successfully observed cellulose microfibrils and matrix polysaccharides after microbial digestion (27) (Fig. S1). To quantitatively evaluate the fiber digestion of pigs, Image Pro Plus software was used to determine the area of residual matrix polysaccharide, the area of damaged cellulose microfibrils, and the area of total fecal particles. Then, the completeness rate of matrix polysaccharide (CRMP: the area of residual matrix polysaccharide/the area of total fecal particle) and the damage rate of cellulose microfibrils (DRCM: the area of damaged cellulose microfibrils/the area of total fecal particle) were defined. To ensure the accuracy of the results obtained, six random microscopic fields of view for each sample were selected for the determination of CRMP and DRCM.

### Shotgun metagenome sequencing and data analysis

Total genomic DNA was extracted from cecal content samples using the E.Z.N.A. Soil DNA Kit (Omega Bio-tek, Norcross, GA, USA) according to the manufacturer’s instructions. Concentration and purity of extracted DNA were determined with TBS-380 and NanoDrop2000, respectively. DNA extract quality was checked on 1% agarose gel.

DNA extract was fragmented to an average size of about 400 bp using Covaris M220 (Gene Company Limited, China) for paired-end library construction. Paired-end library was constructed using NEXTFLEX Rapid DNA-Seq (Bioo Scientific, Austin, TX, USA). Adapters containing the full complement of sequencing primer hybridization sites were ligated to the blunt end of fragments. Paired-end sequencing was performed on Illumina NovaSeq (Illumina Inc., San Diego, CA, USA) at Majorbio Bio-Pharm Technology Co., Ltd. (Shanghai, China) using NovaSeq Reagent Kits according to the manufacturer’s instructions (www.illumina.com).

The paired-end Illumina reads were trimmed of adaptors, and low-quality reads (length <50 bp or with a quality value <20 or having N bases) were removed by fastp (28) (https://github.com/OpenGene/fastp, version 0.20.0). Metagenomics data were assembled using MEGAHIT (29) (https://github.com/voutcn/megahit, version 1.1.2), which makes use of succinct de Bruijn graphs. Contigs with a length being or over 300 bp were selected as the final assembling result, and then, the contigs were used for further gene prediction and annotation. Open reading frames (ORFs) from each assembled contig were predicted using MetaGene (30) (http://metagene.cb.k.u-tokyo.ac.jp/). The predicted ORFs with length being or over 100 bp were retrieved and translated into amino acid sequences using the NCBI translation table (http://www.ncbi.nlm.nih.gov/Taxonomy/taxonomyhome.html/index.cgi?chapter=tgencodes#SG1).

### Determination of fiber degradation substances: polysaccharides, oligosaccharides, and monosaccharides

High-performance gel permeation chromatography (HPGPC) was performed to detect the molecular mass (MM) distribution of the polysaccharides in cecal content. The sample and standard were accurately weighed, and then prepared the sample into 5 mg/mL solution with deionized water. After centrifugation at 12,000 rpm for 10 min, the samples were filtered using 0.22 µm microporous membrane, and then were transferred into a 1.8 mL injection vial for testing. The separation was conducted through a tandem gel column (BRT104-102, 8 × 300 mm, Borui Saccharide, Biotech. Co. Ltd. Jiangsu, China). The injection volume per sample was 20 µL. The eluent was 0.05 M NaCl, eluted at 40°C with a flow rate of 0.6 mL/min. The molecular mass was estimated using a standard curve based on Dextran standards (Sigma, Sigma-Aldrich [Shanghai] Trading Co Ltd, Shanghai, China).

Oligosaccharides analysis was performed by ICS5000 ion chromatography (IC) system (ThermoFisher, Thermo Fisher Scientific, Massachusetts, USA). The separation was done using a DionexCarbopacTMPA200 (3*150) column. Oligosaccharides in samples were eluted with H_2_O, 130 mM NaOH, and 1 M NaOAC in turn, eluted at 30°C with a flow rate of 0.3 mL/min. The Oligosaccharide concentration was calculated using a standard curve based on maltose standards.

A total of 14 monosaccharide standard curves (including fucose, galactosamine hydrochloride, rhamnose, arabinose, glucosamine hydrochloride, galactose, glucose, N-acetyl-D-glucosamine, xylose, mannose, fructose, ribose, galacturonic acid, and glucuronic acid) were built to investigate monosaccharide composition in cecal content. Monosaccharide composition analysis was performed using ICS5000 IC system (ThermoFisher, Thermo Fisher Scientific, Massachusetts, USA). Monosaccharides in samples were eluted with H_2_O, 15 mM NaOH, and 0.1 M NaOAC in turn, eluted at 30°C with a flow rate of 0.3 mL/min.

### SCFA determination

The SCFA concentrations in the content samples of caecum and colon were determined by gas chromatography (GC) as described previously (31). The sample peaks were identified by comparing their retention times with internal standards of acetate, propionate, butyrate, isobutyrate, valerate, isovalerate, and metaphosphoric-crotonic acid.

### RNA extraction, sequencing, and qRT-PCR

The protocol of cecal tissue RNA extraction was as follows. In brief, approximately 100 mg of each sample was taken in a mortar pre-cooled by liquid nitrogen and ground rapidly to powder without obvious particles, then was transferred into a 1.5 mL centrifuge tube. TRIzol (1 mL) (Takara Bio, Otsu, Japan) was added into the tube, and the mixture was incubated at room temperature for 5 min to dissolve the powder and allow completion of cell lysis. After addition of 200 µL of chloroform to the mixture and centrifugation at 12,000 × *g* for 15 min at 4°C, the supernatant was transferred to another 1.5 mL centrifuge tube. Isopropanol (500 µL) was added, then the mixture was centrifuged at 12,000 × *g* for 10 min at 4°C and the supernatant was discarded. 1 mL of 75% ethanol was added to wash the RNA precipitate, then the solution was dried naturally at room temperature for 5–10 min. After the ethanol evaporated, 30 µL of DEPC water was added to dissolve RNA. The obtained RNA was stored at −80°C for testing.

The purity of RNA was detected by NanoDrop spectrophotometer (ND-1000 UV-Vis; Thermo Fisher Scientific, Waltham, MA, USA). After the samples passed the quality inspection, the cecal mRNA was stored at −80°C. One sample in MS-10.5 was excluded in subsequent transcriptome analysis due to its RNA integrity being unqualified. For library construction, the cecal mRNA was enriched with magnetic beads with oligo (DT). Then, fragment buffer was added to break the mRNA into short fragments of 150–200 BP. Then, taking the mRNA as the template, one strand of cDNA was synthesized with six base random primers, and then buffer, dNTPs, and DNA polymerase I were added to synthesize two-strand cDNA. Then, double-strand cDNA was purified with AMPure XP beads. The purified double-stranded cDNA was repaired at the end, added with a tail, and connected with the sequencing connector, then the fragment size was selected with AMPure XP beads, and finally, the final cDNA library was obtained by PCR enrichment. The samples were sequenced by paired-end sequencing through Illumina Hiseq 2500 sequencing platform. read length of Illumina Hiseq 2500 sequencing was 100 bp. Then, 1 µg RNA of each sample was converted into cDNA with the Reverse Transcriptase Kit (Takara, Japan) following the manufacturer’s instructions. Real-time PCR was performed on ABI PRISM 7300 sequence detection system (SDS, Foster City, CA, USA) using SYBR Premix DimerEraserTM Kit (Takara, Japan) following the manufacturer’s instructions. The primer sequences are listed in Table S2.

### Development parameters of cecum intestinal wall

After all pigs were slaughtered, approximately 2 cm × 2 cm of cecal tissue samples were collected from each pig. The cecal contents on the surface of the samples were washed with sterile physiological saline, then samples were loaded with 4% paraformaldehyde for morphological observation. The thickness of cecum mucosa, submucosa, muscularis, and whole intestinal wall was observed after staining with hematoxylin and eosin (HE). These morphometric indices were measured using a Nikon ECLIPSE 80i light microscope with a computer-assisted morphometric system (Nikon Corporation, Tokyo, Japan).

### Morphological observation of cecal epithelium

The main indexes were the density of goblet cells in the cecum epithelium, proliferation and apoptosis of cecum epithelial cells. The combined Alcian Blue/periodic acid Schiff (AB-PAS) stain technique was then employed to measure the cecal goblet cell density (32). In particular, deparaffinized and rehydrated sections were stained with 1% Alcian Blue solution (Alcian Blue in 3% acetic acid solution), gently washed in double-distilled H_2_O for 10 min, oxidized in 1% periodic acid solution for 15 min, rinsed twice with double-distilled H_2_O for 10 min, and then placed in periodic acid Schiff solution for 30 min, rinsed with running water for 5 min. Goblet cells were counted in 12 well-oriented crypts per group. Goblet cell density was calculated as the goblet cell count divided by the corresponding crypt depth, averaged, and expressed as goblet cell number per 100 µm of crypt depth (33).

Ki-67 immunohistochemistry staining was used to determine cecal mucosal crypt cell proliferation (34). First, the slides were treated as antigen retrieval processes. The slides cannot be dried for preventing excessive evaporation of buffer solution during the process. After the slices were incubated with 3% hydrogen peroxide solution at room temperature and avoided light for 25 min to quench endogenous peroxidase, the slides were incubated with primary antibody (Proliferation marker protein Ki-67; Wuhan Servicebio Biotechnology Co., Ltd., Hubei, China; 1:300) diluted in PBS overnight at 4°C. Then, the slides were incubated with the biotinylated goat anti-rabbit secondary antibody for 50 min. Finally, Di-amino-benzidine (DAB) chromogenic reagent was used to control the desired stain intensity. Twelve random crypts in each slice were analyzed. The total number of crypt cells and the number of Ki-67 positive cells (brown cells) in each crypt were counted to calculate the percentage of proliferative cells.

We then detected the percentage of apoptotic cells in the cecum of pigs using terminal deoxynucleotidyl transferase-mediated deoxyuridine triphosphate nick end labeling (TUNEL) staining (35). The slides were rehydrated as follows: Soaked the slices in xylene for 10 min, and then soaked for 10 min after replacing xylene. Soaked with 100% ethanol, 95% ethanol, 85% ethanol, and 70% ethanol in order, following a PBS washing three times. Then, the slides were incubated with 10× Proteinase K for 15–20 min at 37°C. The slides were placed in the H_2_O_2_ solution (3% in methanol) for 20 min at 25°C to deactivate endogenous peroxides, following a PBS washing three times. After that, the slides were incubated with terminal deoxynucleotidyl transferase (TDT) for 60 min at 37°C, following a PBS washing three times. The slides were incubated with horseradish peroxidase-conjugated streptavidin solution (Streptavidin-HRP) for 30 min at 37°C, following a PBS washing three times. After that, the slides were incubated with DAB solution and Hematoxylin solution in turn. The number of TUNEL-positive cells (brown cells) at every 200 cecal epithelium cells in seven random mucosal areas per slide were counted to calculate the percentage of apoptotic cells.

Slides of cecal epithelium are scanned in all directions using an award-winning all-round digital slide scanner (PANNORAMIC MIDI II from 3DHistech Kft., Hungary). Morphological observations and measurements were realized using SlideViewer 2.5.0.

### Statistical analysis

The data were first initially organized through Excel 2019. Subsequently, statistical analysis was performed by using SPSS 20.0. Polynomial contrasts were conducted to determine the effects of the increasing dietary fiber level on growth performance and SCFAs using SPSS 20.0. For comparing the data of microbial relative abundance, Mann-Whitney U test was performed for pairwise comparisons. The visualization of regular statistics and correlation heat map and bubble map are mainly implemented by Origin 2023. The visualization of microbial co-occurrence network analysis was conducted using Cytoscape 3.9.0 (36). Visualization of phylogenetic analysis was performed using Chiplot (37) (https://www.chiplot.online/). The screening of candidate biomarkers from metagenome, and tissue transcriptome was based on the ATTD of MS and LW. Briefly, microbial species and host genes, which had no significant difference between MS-CON and MS-10.5, and had significantly lower abundance in LW-10.5 than those in LW-CON, were filtered to resolve potential mechanisms for the difference in fiber utilization between MS and LW. Spearman correlation coefficient is used to measure the link of gut genes, microbes, and SCFAs using SPSS 20.0. Statistical significance was defined as *P* < 0.05.

## RESULTS

### Digestion and utilization of high-fiber diets in pigs vary with breeds

**Fig 1:**
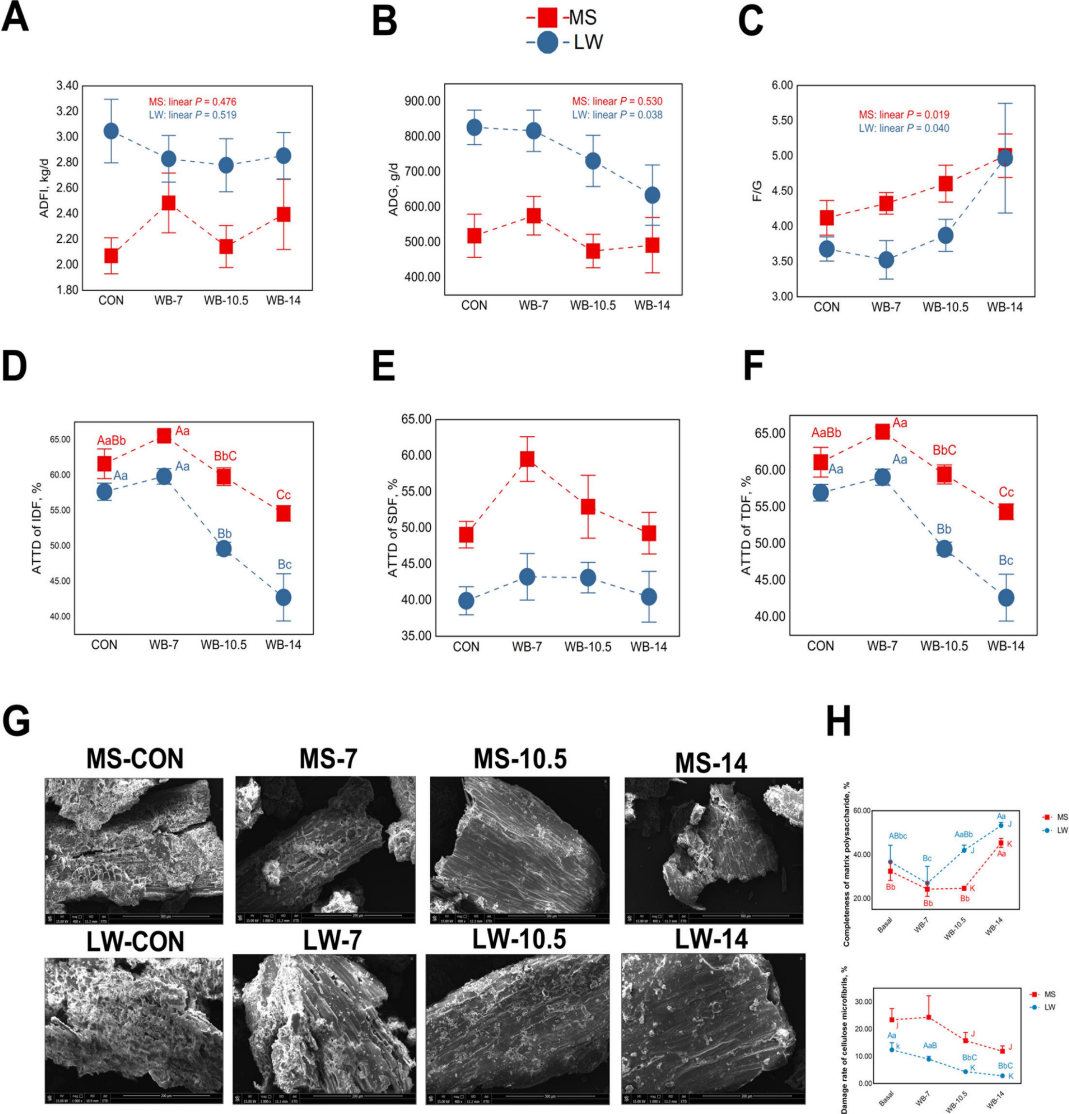
Responses of growth performance and fiber digestion of pigs to dietary fiber level. (**A-C)** Growth performance average daily feed intake (ADFI), average daily gain (ADG), and feed-to-gain ratio (F/G). Linear changes were used to indicate the extent to which the indicator was affected by fiber. (**D-F)** Apparent total tract digestibility (ATTD) of dietary fiber. (**G)** The microstructure of dietary fiber fermented by gut microbiota was obtained through scanning electron microscopy (SEM), with observations conducted at scales of 200 or 500 µm, respectively. Different uppercase letters indicate *P* < 0.01, and different lowercase letters indicate *P* < 0.05. (**H)** Quantification of the completeness rate of matrix polysaccharide (CRMP) and the damage rate of cellulose microfibrils (DRCM). Different uppercase letters indicate *P* < 0.01, and different lowercase letters indicate *P* < 0.05. A, b and c are used to measure differences in fiber levels, whereas j, k, and l are used to measure differences between MS and LW.

With increasing WB levels, F/G of MS and LW both increased (linear *P* < 0.05), and ADG of LW decreased (linear *P* < 0.05), but ADG of MS was not changed (Fig. 1A through C). In MS, ATTD of IDF and TDF in WB-10.5 was not significantly impaired but significantly decreased in WB-14 (*P* < 0.01), compared with the CON. Although in LW, ATTD of IDF and TDF of WB-10.5 and WB-14 significantly decreased (*P* < 0.01), compared with the CON (Fig. 1D through F).

The clear microstructure of dietary fiber fermented by microbiota was obtained by SEM analysis (Fig. 1G), CRMP of MS and LW in WB-14 was higher than that in the CON (*P* < 0.05). Notably, in high-fiber diets (WB-10.5 and WB-14), CRMP of MS was lower than that of LW (*P* < 0.01). DRCM of MS was not affected by dietary fiber level, whereas DRCM of LW in WB-10.5 and WB-14 was lower than those in the CON (*P* < 0.05) (Fig. 1H). Moreover, in high-fiber diets (WB-10.5 and WB-14), DRCM of MS was higher than that of LW (*P* < 0.01) (Fig. 1H).

To sum up, the difference in fiber-digested capability between the two pig breeds was obviously reflected in the high fiber diet (WB-10.5), based on the different ATTD of dietary fiber and microstructure of feces between MS and LW. Therefore, samples from pigs in MS-CON, MS-10.5, LW-CON, and LW-10.5 (28 in total) were selected for multi-omics analysis.

### Display of cecal microbial composition in MS and LW

**Fig 2:**
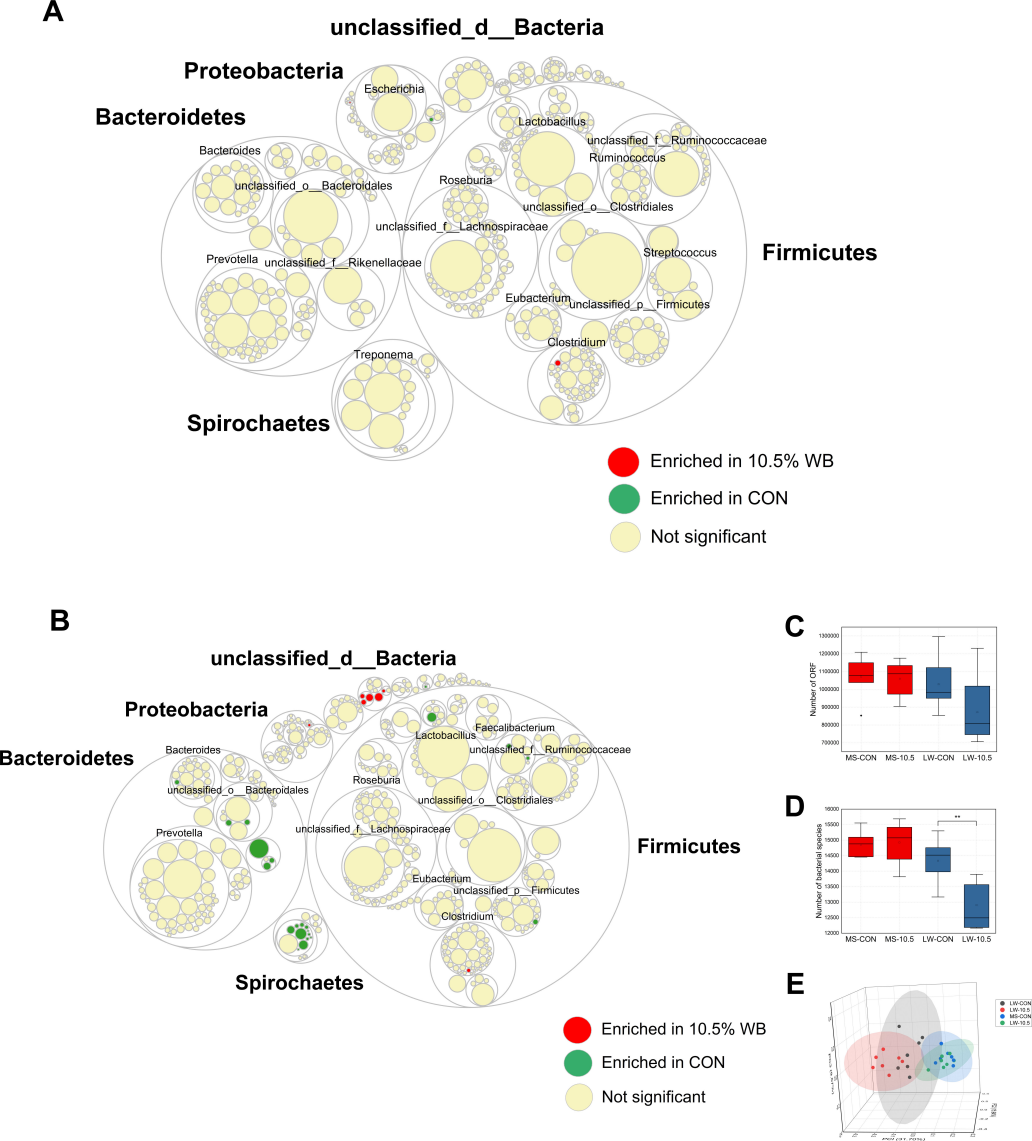
Responses of cecal microbiota of Meishan pigs and Large White pigs to dietary fiber. (**A** and **B)** Displayed are cecal microbial composition of Meishan pigs and Large White pigs (relative abundance in top 500), respectively. Round nested diagram shows, from the outside to the inside, the phyla, families, genera, and species. Red filled circles indicate species enriched in the WB-10.5 (*P* < 0.01), green filled circles indicate species enriched in the CON (*P* < 0.01), and yellow filled circles indicate species that are not significantly different between the control and WB-10.5 groups. (**C** and** D)** Comparison of ORF numbers and species numbers. (**E)** Principal coordinate analysis based on Bray_Curtis distance at the species level of MS-CON, MS-10.5, LW-CON, and LW-10.5.

A total of 417.44 Gb metagenome sequencing data were obtained from 28 samples, with 14.91 Gb data per sample. After quality control of raw data and removal of contaminated sequence, 285.57 Gb optimized reads were spliced into contigs, then the open reading frame (ORF) were predicted using MetaGene (30) (http://metagene.cb.k.u-tokyo.ac.jp/). As a whole, increasing of dietary fiber level did cause very few significant changes in the cecal microbial composition of MS but greatly changed cecal microbial composition of LW (Fig. 2A and B), which is further supported by the principal coordinate analysis (ANOSIM, *R* = 0.29, *P* < 0.01, Fig. 2D). In particular, the members of Bacteroidetes and Spirochaetes of LW-10.5 had lower relative abundance than those in LW-CON (Fig. 2B). The numbers of bacterial species in MS were not affected by WB, whereas the numbers of bacterial species in LW-10.5 were lower than those in LW-CON (*P* < 0.01) (Fig. 2C). Subsequently, the species-level microbes that present in all cecum contents samples from MS-CON and MS-10.5, and the relative abundance was significantly (*P* < 0.01) lower in LW-10.5 than those in LW-CON. Based on this candidate microbial screening approach, 181 species-level microorganisms were initially screened (Table S3). Then, these 181 candidate biomarkers would be used to investigate their fiber-degrading potential.

### Breed-specific fiber-degrading potential of microbiota

A total of 5,392,222 non-redundant genes were obtained from metagenome data. To investigate fiber-degrading related functions of cecal microbiota in MS and LW, the non-redundant genes were hunted against the KEGG (Kyoto Encyclopedia of Genes and Genomes) database and CAZymes database (Carbohydrate-active enzymes database). Of the non-redundant genes, 19.45% were successfully annotated to KEGG database, and 3.42% were successfully annotated into CAZymes database.

To further explore the functional potential of candidate microbes for fiber degradation, we further analyzed the microbial information annotated into CAZymes database. At class level, annotated glycoside hydrolases (GHs) in all pigs were the most (41.1%–44.8%), followed by glycosyl transferases (GT: 34.3%–38.7%), carbohydrate esterases (CE: 13.7%–14.1%), carbohydrate-binding modules (CBM: 3.0%–3.4%), auxiliary activities (AA: 2.1%–2.6%), and polysaccharide lyases (PL: 0.7%–1.3%) (Fig. S2A). The enzymes in GHs could cleave glycosidic bonds between carbohydrates or between a carbohydrate and a non-carbohydrate moiety, which play an important role in fiber degradation (38, 39). Notably, the expression of enzymes in main GH families (top 20) has breed-heterogeneity, and the GH families in MS generally have higher abundance, compared with those in LW. Compared with pig breeds, the diets seemed to have fewer effects (Fig. S2B).

**Fig 3:**
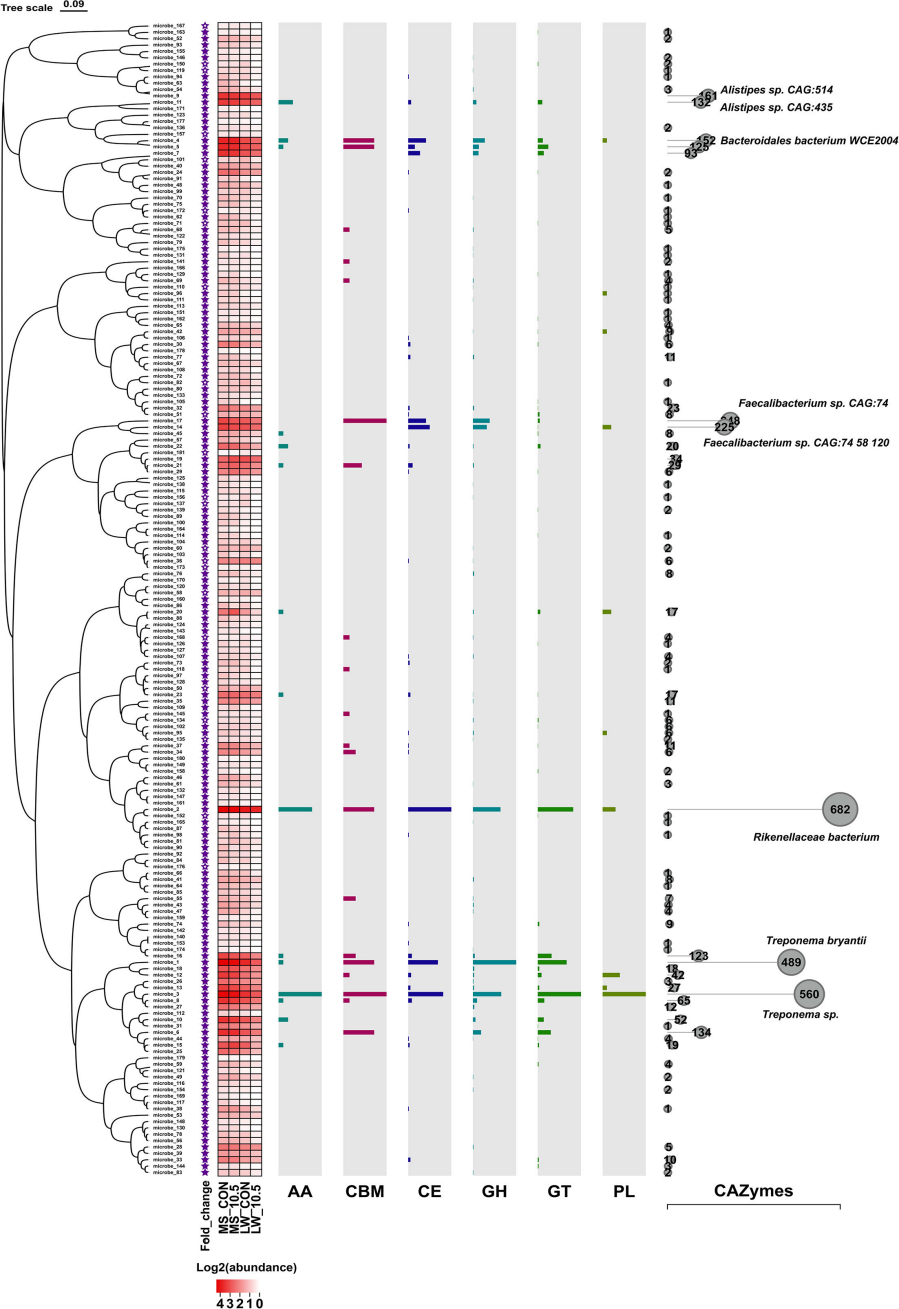
Carbohydrate-active enzyme (CAZyme) properties of a specific gene set (SGS) constructed from 181 candidate species-level microbes. The species names corresponding to the microbial numbers are shown in Table S4. Solid pentagrams indicate fold change of microbial relative abundance >1 (MS/LW >1), and hollow ones indicate fold change <1 (MS/LW <1). The heat map shows the microbial abundance in the four groups. The bar graphs indicate the difference in the number of genes encoding carbohydrate-active enzymes for each microbe (the value = MS minus LW). AA, CBM, CE, GH, GT, and PL denote auxiliary activities, carbohydrate-binding module, carbohydrate esterase, glycoside hydrolase, glycosyl transferase, and polysaccharide lyase, respectively. The total number of genes encoding CAZymes per microbe is shown using lollipop plots.

To screen specific microbes that may be related to the difference of fiber degradation between MS and LW, the genes of the candidate biomarker (the 181 bacterial species) were used to construct a specific gene set (SGS). Carbohydrate-active enzyme (CAZyme) properties of the SGS were further investigated (Fig. 3). In SGS, the number of CAZymes-encoding genes of most microbes was higher in MS than those in LW. Moreover, after counting the total number of CAZymes for each species, we found that *Alistipes* sp. CAG:435, *Alistipes* sp. CAG:514, *Bacteroidales* bacterium WCE2004, *Rikenellaceae* bacterium, *Faecalibacterium* sp. CAG:74, *Faecalibacterium* sp. CAG:74 58 120, *Treponema bryantii*, and *Treponema* sp. contained the highest number of CAZymes-encoding genes, accounting for 70.51% of CAZymes-encoding genes of SGS. GH was the most important enzyme for microbial degradation of dietary fiber; hence, we further investigated the major species possessing GH-encoding genes in SGS, and similarly, these eight microbes possessed a very high number of various GH-encoding genes, accounting for 83.50% of GH-encoding genes of SGS (Fig. S2C). After investigating CAZyme characteristics of 181 candidate microbes, these eight species were identified as important functional species for elucidating the differences in fiber utilization efficiency between MS and LW.

**Fig 4:**
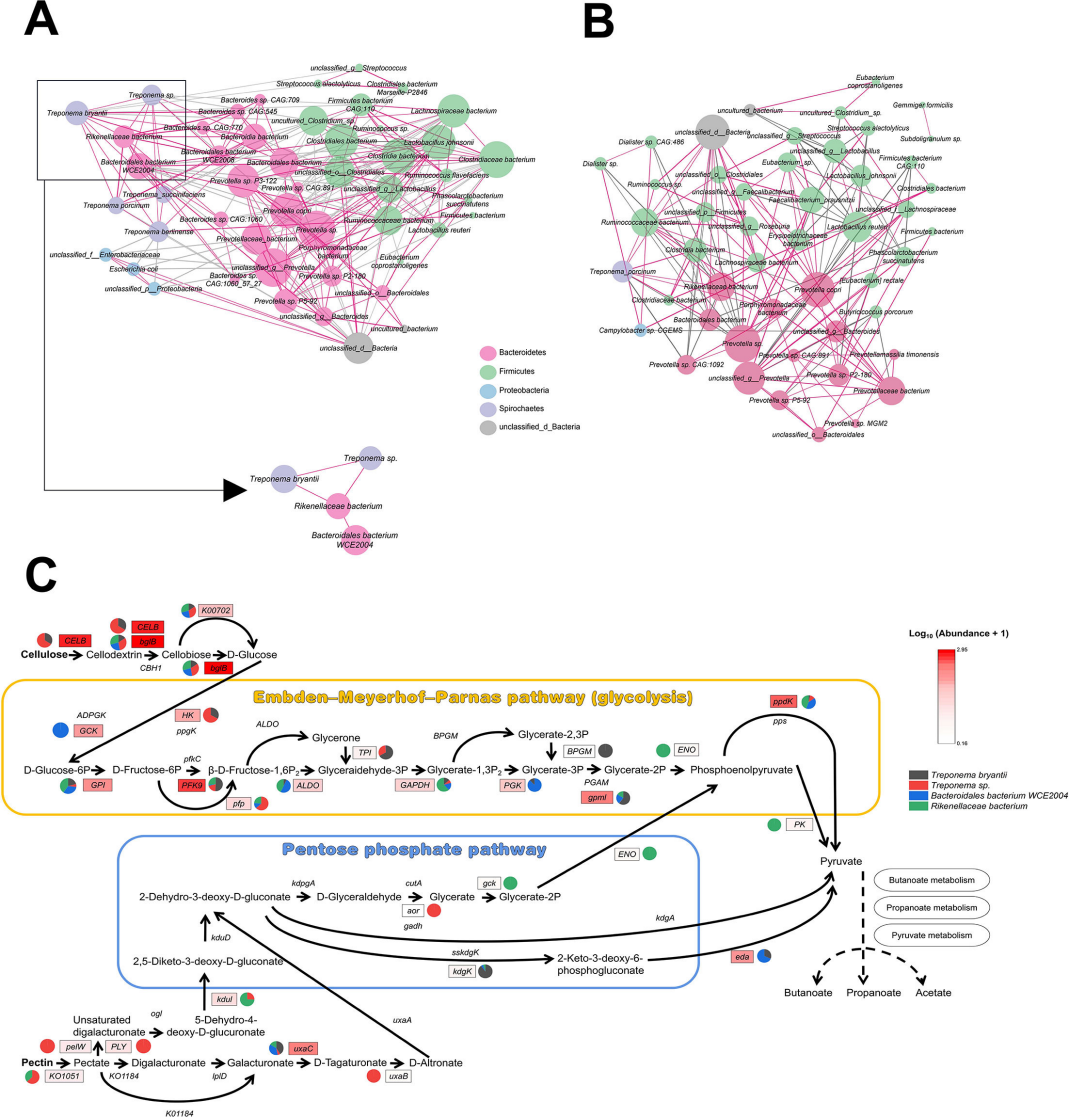
The interactions of cecal microbiota during fiber degradation. (**A)** Co-occurrence network analysis of cecal microbes in MS. (**B)** Co-occurrence network analysis of cecal microbes in LW. The different colored nodes indicate the microbes of different phyla, and the node size indicates the relative abundance of microbes. The red lines indicate a significant positive correlation (*P* < 0.05), and the gray lines indicate significant a negative correlation (*P* < 0.05). (**C)** Division and cooperation of key microbes in the degradation of cellulose and pectin to monosaccharides and then involved in the production of SCFAs. The enzymes successfully annotated were highlighted with rectangular boxes, and the filling color of the rectangular box indicates the abundance of the enzyme. The pie chart shows the proportion of enzymes encoded by four species.

The intestinal microbiota is a vast, intricate assembly, and microbial interactions significantly affect feed digestion (40). Therefore, we next performed a microbial co-occurrence network analysis in which it is noteworthy that *Rikenellaceae* bacterium, *Bacteroidales* bacterium WCE2004, *Treponema bryantii,* and *Treponema* sp. had significant positive relationships in MS (Fig. 4A), whereas these relationships were not found in LW (Fig. 4B). KEGG functional annotations of these four species were performed to explore specific interactions of these four species in the process of dietary fiber degradation (Fig. 4C). A total of 27 kinds of enzymes were annotated to these four species during the degradation of cellulose and pectin to monosaccharides and then involved in the production of SCFAs through the Embden–Meyerhof–Parnas pathway (EMP) pathway and pentose phosphate pathway, respectively (Fig. 4C). Among them, the top five enzymes with the highest abundance were *bglB* [EC: 3.2.1.21], *CELB* [EC: 3.2.1.4], *PFK9* [EC: 2.7.1.11], *ppdK* [EC: 2.7.9.1], and *gpml* [EC: 5.4.2.12] in order. *bglB* degraded cellulose to cellodextrin, and then, cellodextrin was degraded to cello-disaccharide and monosaccharide in one step under the action of *CELB. PFK9*, *ppdK,* and *gpml* were the key enzyme in EMP pathway. Also, important enzymes related to pectin degradation were annotated through these four species. In addition, these four species have diverse forms of division of labor in cellulose and pectin degradation, for example, in the process of cellulose to monosaccharide, all four species encode *bglB* and *KO0702* (cellobiose phosphorylase [EC:2.4.1.20]), whereas *Treponema bryantii* and *Treponema* sp. were the main contributors of *CELB*. In the process of pectin to monosaccharide, most of enzymes were encoded by *Treponema bryantii* and *Rikenellaceae* bacterium, such as *KO1051* [EC: 3.1.1.11], *PLY* [EC: 4.2.2.2], *uxaB* [EC: 1.1.1.58], and *kdul* [EC: 5.3.1.17] (Fig. 4C).

### Distribution of fiber-degrading substances in cecum of MS and LW

**Fig 5:**
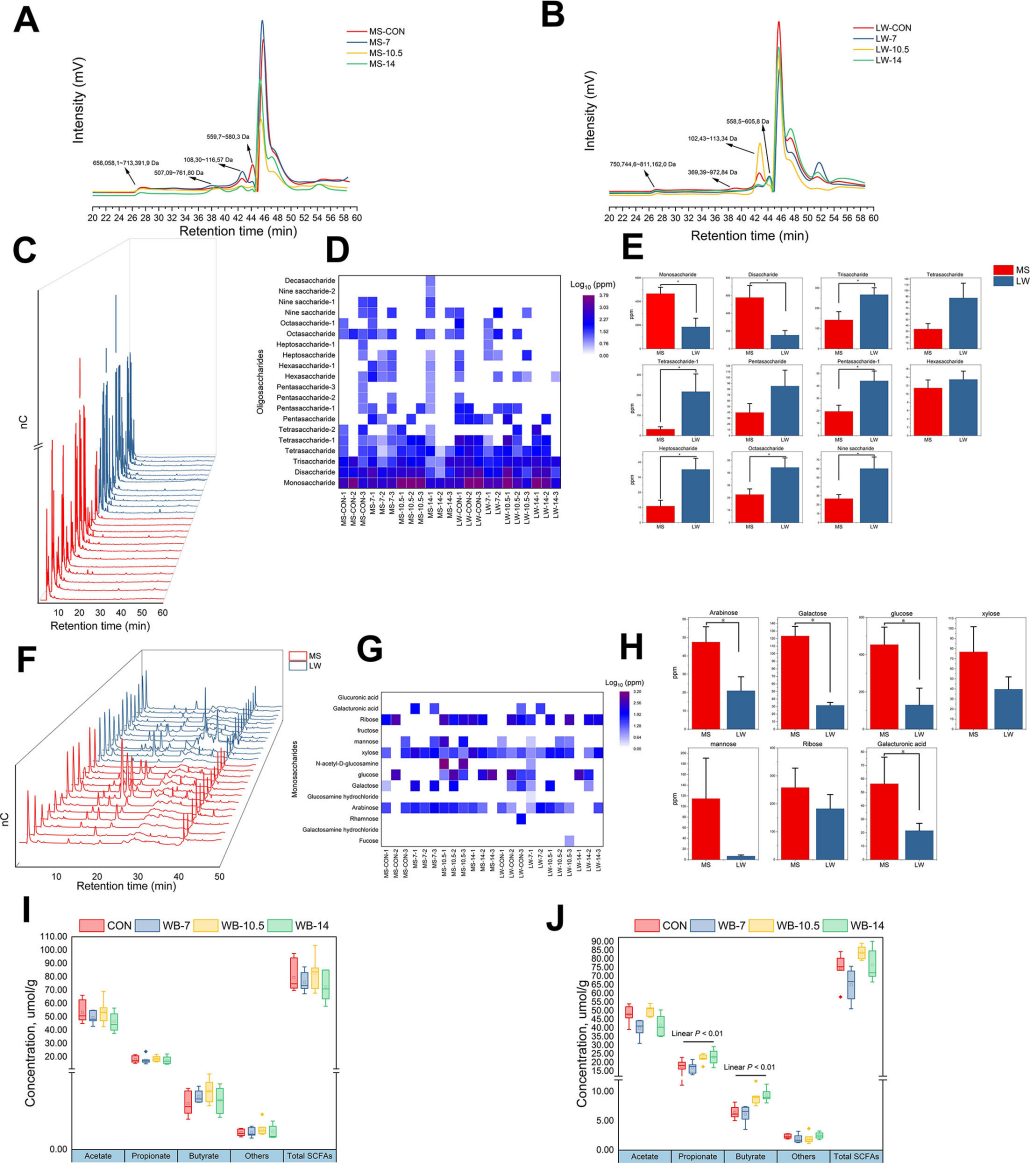
Fiber-degrading products at various levels of pig cecal contents. (**A** and **B)** Distribution of molecular weight of polysaccharides in cecal contents of MS and LW. (**C-H)** Comparisons of concentrations of oligosaccharides (monosaccharide ~decasaccharide) and 14 specific monosaccharides in cecum content between MS and LW. (I) and (J) SCFA content in cecum contents of MS and LW. * means *P* < 0.05, ** means *P* < 0.01.

The degradation of dietary fiber by gut microbiota first involves the action of microbial CAZymes, which gradually cleave high molecular weight and highly polymerized fiber into monosaccharides. Then, monosaccharides are the main substrates for subsequent reactions (15). Therefore, after evaluating fiber-degrading potential of cecal microbiota in MS and LW, HPGPC and IC analyses were used to further investigate the actual situation of fiber degradation in two pig breeds. First, HPGPC analysis revealed that the molecular weight (mm) of polysaccharides was mainly distributed in four sections, including 6,560,581–7,133,919 Da, 50,709–76,180 Da, 10,830–11,657 Da, and 5,597–5,803 Da in MS, 7,507,446–8,111,620 Da, 36,939–97,284 Da, 10,243–11,334 Da, and 5,585–6,058 Da in LW (Fig. 5A and B). Subsequently, IC analysis detected oligosaccharides of different sizes (containing 1–10 monosaccharide molecular) (Fig. 5C and D). Among them, oligosaccharides (1–4 monosaccharide molecular) were common in most samples and the greater the molecular mass, the less concentration of oligosaccharides in cecum (Fig. 5D). Interestingly, concentrations of monosaccharides and disaccharides in the cecum of MS were significantly higher than those of LW (*P* < 0.05, Fig. 5E), whereas concentrations of other oligosaccharides (3, 5, 7, 8, and 9 monosaccharide) were significantly lower than those of LW (*P* < 0.05, Fig. 5E). Then, 14 kinds of monosaccharides were detected by IC (Fig. 5F and G). The results revealed arabinose, galactose, glucose, xylose, and ribose were common in most samples (Fig. 5G). Among them, concentrations of arabinose, galactose, glucose, and galacturonic acid in MS were higher than those of LW (Fig. 5G). Finally, concentrations of SCFAs, the end products of dietary fiber digested by microbiota, were detected by GC. The results showed there was no difference among diets in MS (Fig. 5I), whereas concentration of propionate and butyrate increased with the increasing of dietary fiber in LW (*P* < 0.01, Fig. 5J).

### Integrative analysis of host intestine, cecal microbes, and SCFAs

**Fig 6:**
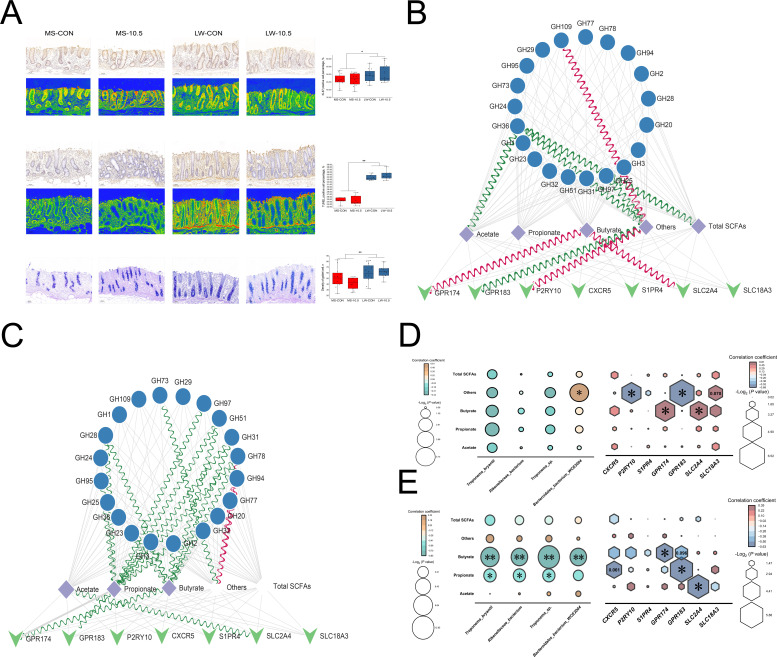
Cecum mucosal morphology and host-microbe interaction analysis. (**A)** Ki-67 staining, TUNEL staining of cecal mucosa and AB-PAS staining of goblet cells on cecal mucosa. Each slice was provided a regular scanning view and a gradient map. * means *P* < 0.05, ** means *P* < 0.01. (**B** and **C)** Interaction network among host genes, microbial CAZymes (TOP 20 GHs), and cecal SCFAs in MS and LW. Red wavy lines indicate a positive correlation (*P* < 0.01), green wavy lines indicate a negative correlation (*P* < 0.01), and gray straight lines indicate no significant correlation. (**D** and **E)** Interaction among host genes, microbes, and cecal SCFAs in MS and LW. * means *P* < 0.05, ** means *P* < 0.01.

Regarding why SCFA concentration remained steady in MS but rose in LW, we analyzed from two angles: intestinal structure and SCFA transport gene expression. We first implemented a histomorphometric observation of cecum, then found that overall thickness of cecum in Meishan pigs is higher than that in Large White pigs by HE staining (*P* < 0.01, Fig. S3). However, dietary fiber did not cause differences in cecum epithelial morphology, but there were significant differences in cecum epithelial morphology (densities of goblet cells, Ki-67 positive cell percentage and TUNEL positive cell percentage) between MS and LW (*P* < 0.05, Fig. 6A). RNA-seq was conducted to explore adaptive alterations in intestinal gene expression at the transcriptome level with rising dietary fiber. After data quality control, a total of 75.6 Gb clean data were obtained. According to the method of biomarker gene screening in this study (based on the ATTD of MS and LW). A total of 166 candidate marker genes were obtained in cecum (LW-10.5 vs LW-CON: FDR < 0.05; |Log_2_ (fold change)| >1, and no difference between MS-10.5 and MS-CON). Among 166 candidate marker genes, there were 52 upregulated genes (*P* < 0.01, Table S5) and 114 downregulated genes in LW-10.5 (*P* < 0.01, Table S6). KOBAS was performed to conduct GO (Gene Ontology) enrichment analysis of 166 candidate marker genes, and a total of 120 GO terms were enriched. SCFAs produced by microbial-digested fibers can be recognized and absorbed by large intestine epithelial cells (41). Therefore, genes related to cell membrane composition were specially screened. In cellular component term, there were eight GO terms related to cell membrane composition (Fig. S4A). *CXCR5*, *P2RY10*, *S1PR4*, *GPR174*, *GPR183*, *SLC2A4,* and *SLC18A3* were involved in cell membrane composition.

To comprehend the impact of breed-heterogeneity relations between host genes and intestinal microbiota on SCFA absorption, interaction network analyses among host genes (*CXCR5*, *P2RY10*, *S1PR4*, *GPR174*, *GPR183*, *SLC2A4,* and *SLC18A3*), specific microbes (*Rikenellaceae* bacterium, *Bacteroidales* bacterium WCE2004, *Treponema bryantii,* and *Treponema* sp.), glycoside hydrolases (GHs), and cecal SCFAs were carried out separately (Fig. 6A through D). In MS, *GPR174* and *SLC2A4* were positively correlated with butyrate (*P* < 0.01, Fig. 6B and D). In LW, *SLC2A4*, *GPR183,* and *GPR174* were negatively correlated with acetate, propionate, and butyrate, respectively (*P* < 0.01, Fig. 6C and E). In addition, concentrations of propionate and butyrate in cecum of LW were almost negatively correlated with *Rikenellaceae* bacterium, *Bacteroidales* bacterium WCE2004, *Treponema bryantii,* and *Treponema* sp. (*P* < 0.05), no similar results were found in MS. Finally, expression of *SLC2A4*, *GPR183,* and *GPR174* was validated using quantitative PCR, and the expression trends remained consistent (Fig. S4B).

## DISCUSSION

High-fiber feed ingredients, due to their anti-nutritional properties, have very limited application in livestock production (5). It has been well documented that Meishan pig, as a local pig breed in China, has a higher tolerance to dietary fiber relative to commercial pigs, mainly in terms of higher ATTD of fiber (7, 8, 42). Similar to previous studies, the present study found that ADG of Meishan pigs almost did not be affected by high-fiber diets. The ADG of LW decreases with the increasing of fiber. Although the most direct reason is that LW cannot tolerate high fiber levels in their diet, the impact of lower nutritional levels on their ADG cannot be ignored. Therefore, it is necessary to explore the effect of high fiber on the growth performance of LW under conditions that fully meet their nutritional needs in the future. The ATTD of IDF and TDF of Meishan pigs could be maintained without decreasing under high-fiber diet (10.5% wheat bran replacement). Based on this, our study revealed the microstructure of residual fibers in pig feces via SEM, finding notable differences in matrix polysaccharide (26, 43) and cellulose degradation between MS and LW. A microscopic view was provided for the investigation of the digestive status of dietary fiber in pigs. Observing microstructure of residual fibers in pig fecal samples is convincing and creative for describing fiber digestion in pigs. These findings directly reflect the higher fiber-digested capability of Meishan pigs to high-fiber diets. Fiber degradation by hindgut microbiota is the sole channel for monogastric animals to utilize dietary fiber (15), and CAZymes of fiber-degrading microbiota are dominated in the process of microbial fiber degradation (44). Therefore, a study of intestinal microbiota and its functional profiles is of great interest to explain the mechanism of high fiber diet tolerance in pigs.

Numerous microbiome studies have found that swine gut microbiota are closely related to feed utilization efficiency (45), gut development (46), fat deposition (47), growth performance (48), and that the genomic functions of gut microbes can be said to complement those of the host genome (49). One of the most important complementary functions is the ability of the microbial genome to encode carbohydrate-active enzymes to degrade dietary fibrous materials, which is not achievable with human endogenous digestive enzymes (15). In this study, we investigated the cecal microbial community of Meishan and Large White pigs using metagenome sequencing and found some interesting phenomena: the amount of microbial community in Meishan pigs was not affected by high-fiber diets, but high-fiber diet reduced population size and altered cecum microbial composition and functions of Large White pigs. Dietary fiber is an important shaper of intestinal microbiota (50, 51); however, the cecal microbial community of Meishan pigs in this study was not affected by high fiber diet. This result indicates that the cecal microbial community of Meishan pigs is more stable than that of Large White pigs. GHs (39) and PLs (52) were able to cut the glycosidic bonds between carbohydrates or between carbohydrate and non-carbohydrate fractions to achieve fiber degradation. In this study, the relative abundance of GH class and PL class in Meishan pigs was higher than that of Large White pigs. To sum up, our results indicated that the cecum microbiota of Meishan pigs had a stronger fiber-degrading potential relative to Large White pigs, which might result in the higher ATTD of IDF and TDF of Meishan pigs than Large White pigs.

Specific fiber-degrading species with pig breed-heterogeneity may be the main contributors to the differences in fiber degradation of two pig breeds. In this study, eight species-level microbes possessing multiple GH family members, including *Alistipes* sp. CAG:435, *Alistipes* sp. CAG:514, *Bacteroidales* bacterium WCE2004, *Rikenellaceae* bacterium, *Faecalibacterium* sp. CAG:74, *Faecalibacterium* sp. CAG:74 58 120, *Treponema bryantii*, and *Treponema* sp*.* were identified among 181 candidate species. Among them, *Treponema* genera were a class of fiber-degrading bacteria found in rumen (53). *T. bryantii* was first isolated and cultured in rumen fluid and was found to act in conjunction with cellulolytic bacteria *F. succinogenes* for cellulose degradation (13). Quan et al. found that *T. bryantii* may be associated with higher feed conversion in pigs by metagenome sequencing (54). Liu et al. found a higher abundance of GH9-encoding gene of *Alistipes* sp. CAG:514 in fecal samples from pigs with high ATTD of ADF using metagenome sequencing, and his study suggested that *Alistipes* sp. CAG:514 may be an unreported microbe with fiber degradation ability (55). Not only that, within these eight species, we found a clear division of labor and positive collaboration among *T. bryantii*, *Treponema* sp., *Rikenellaceae* bacterium*,* and *Bacteroidales* bacterium WCE2004 in the degradation of cellulose and pectin. In summary, these findings reveal the traits of cecum microbiota and fiber-degrading capacity in MS and LW. Additionally, the synergistic effects of fiber-degrading microbes play a crucial role in pig digestion, unattainable by single microbes.

After investigating the fiber-degrading potential of the cecum microbiota, we examined the substances of dietary fiber after degradation by microbiota to assess the actual degradation of dietary fiber by intestinal microbiota in different pig breeds. The plant cell wall, a fibrous constituent, is indigestible by monogastric animal enzymes but can progressively break down into polysaccharides, oligosaccharides, and monosaccharides, (56) and finally metabolized to SCFAs (57) by EMP pathway and Pentose phosphate pathway from hindgut microbiota. In this study, we conducted a comprehensive comparison of fiber substances with different degrees of polymerization in cecum of Meishan pigs and Large White pigs. The molecular weight of the largest polysaccharide in cecum contents of Meishan pigs was smaller than that of Large White pigs. Moreover, the concentrations of monosaccharides and disaccharides in cecum contents of Meishan pigs were higher than those of Large White pigs, whereas concentrations of other larger molecular weight oligosaccharides were lower than those of Large White pigs. The above results showed that cecum microbiota of Meishan pigs possessed stronger fiber-degrading potential than Large White pigs and thus degraded fiber more completely.

When considered collectively, these results indicated that Meishan pigs’ cecal microbial makeup remains unaffected by a high-fiber diet (WB-10.5), and fiber-degrading species’ beneficial interactions facilitate fiber digestion, thereby preventing a decline in fiber ATTD. However, the cecal microbial richness of Large White pigs was reduced by high-fiber diet and the interactions between fiber-degrading species disappeared, which might cause decreasing in fiber ATTD of Large White pigs in high-fiber diet (WB-10.5).

Notably, results of fiber ATTD measurements, chromatographic analysis, and SEM observations indicated that cecal microbiota of Meishan pigs possessed stronger fiber degradation and digested more dietary fiber than Large White pigs. However, the concentration of propionate and butyrate in cecum contents of Meishan pigs did not change with increasing of dietary fiber level, in contrast, concentration of propionate and butyrate in cecum contents of Large White pigs increased. Therefore, we hypothesized that the absorption and utilization efficiency of SCFAs by the cecum epithelium of two pig breeds were different resulting in the different concentrations of SCFAs in cecum contents. With the wide application of RNA-seq in the field of intestinal function studies, candidate molecules for anion-exchange-dependent non-anion-exchange non-dependent SFCAs transport, including the SLC26A, SLC16A, SLC21A, SLC22A, SLC4A, and SLC5A families were identified (16, 58). In this study, RNA-seq found that in high-fiber diet (WB-10.5), *GPR183*, *GPR174,* and *SLC2A4* were stably expressed in cecum of Meishan pigs but decreased in Large White pigs.

Finally, the correlation analysis among fiber-degrading bacteria, SCFAs, and candidate genes revealed that in Large White pigs, relative abundance of fiber-degrading bacteria and expression of *GPR183*, *GPR174,* and *SLC2A4* were negatively correlated with concentrations of butyrate and propionate to varying degrees, respectively, whereas expressions of *GPR174* and *SLC2A4* were positively correlated with concentrations of butyrate in Meishan pigs. These results suggested that expression of *GPR174*, *GPR183,* and *SLC2A4* under high-fiber diets may promote the uptake and transport of SCFAs from cecum contents by intestinal wall, thus facilitating the process of fiber degradation. SCFAs, especially butyrate, are absorbed by intestinal wall and undergo catabolism in intestinal epithelial cells (17), which providing energy for the vital activity of intestinal epithelial cells (59). In the present study, the thickness of the cecal submucosa and whole intestinal wall of Meishan pigs was higher than that of Large White pigs. Therefore, combined with previous studies, it was very likely that cecum of Meishan pigs absorbed more butyrate than that of Large White pigs and used butyrate as a substrate to provide energy for the host itself.

### Conclusion

**Fig 7:**
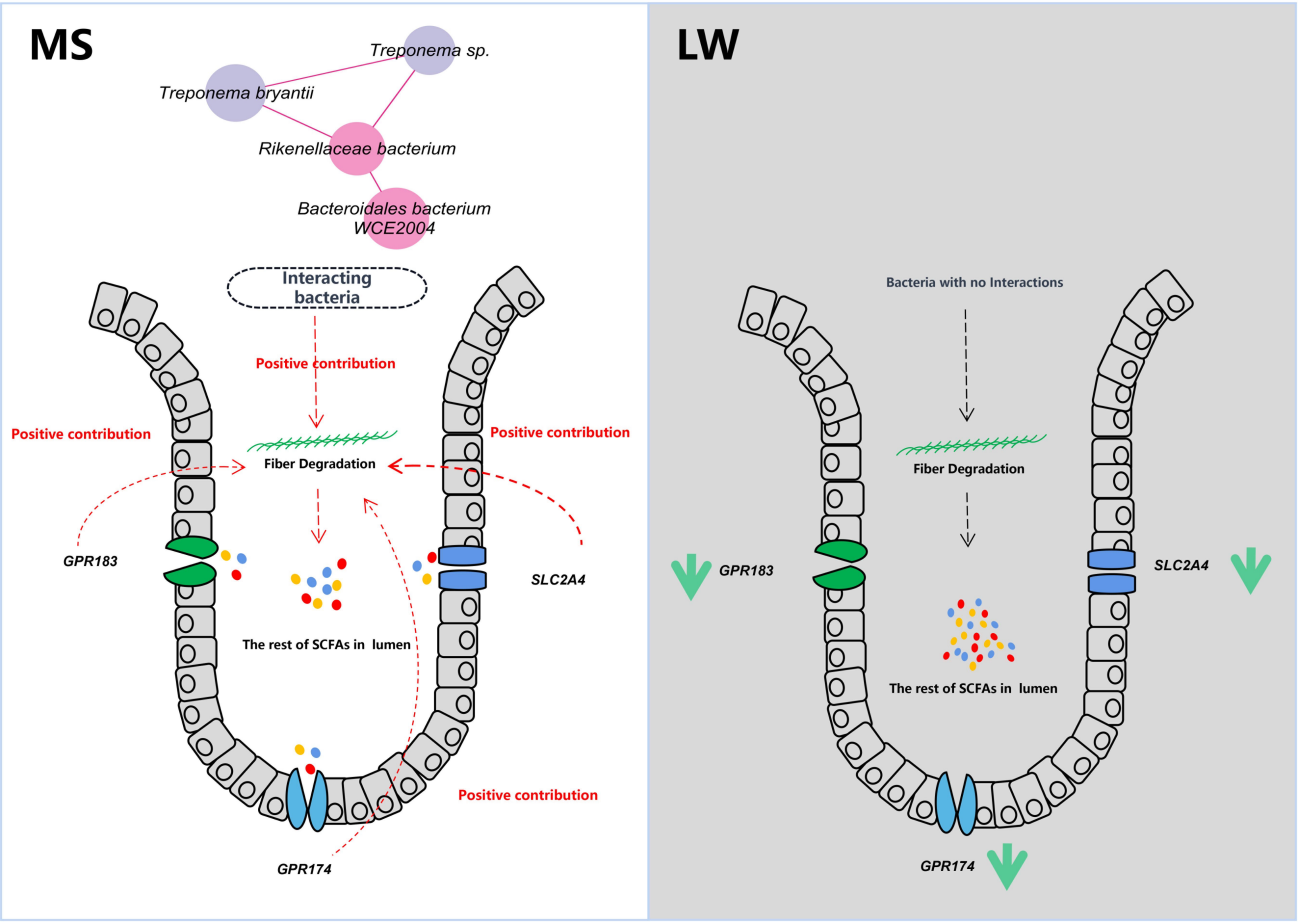
Host-microbiota interactions of pigs during fiber degradation.

The marked disparity in fiber-digestion capabilities between MS and LW under a high-fiber diet (WB-10.5) is evident from their differing ATTD of dietary fiber and fecal microstructure. Metagenomic sequencing illuminated that cecal microbial composition and fiber-degrading functionality of MS remained robust, unhampered by the high-fiber challenge. Notably, key microbial species including *T. bryantii*, *Treponema* sp., *Rikenellaceae* bacterium, and *Bacteroidales* bacterium WCE2004, enriched in GH families, collaborated effectively to enhance cellulose, and pectin degradation in MS (Fig. 7). The detection of polysaccharides and oligosaccharides with reduced degrees of polymerization in the MS’ cecum underscores the superior fiber-degrading proficiency of their cecal microbiota compared with LW. Furthermore, an integrated omics approach disclosed intricate correlations: in Large White pigs, the abundance of fiber-degrading bacteria and expression of *GPR183*, *GPR174*, and *SLC2A4* inversely associated with butyrate and propionate levels, whereas in MS, *GPR174* and *SLC2A4* expressions positively correlated with butyrate concentrations (Fig. 7). These revelations offer fresh perspectives on the mechanisms governing host-microbe interactions that modulate dietary fiber digestion. Importantly, the identified fiber-degrading microorganisms can potentially serve as beneficial strain donors for microbial transplantation, enhancing ATTD of dietary fiber in target pig populations and, consequently, optimizing the utilization of high-fiber feed resources in pig production.

## Data Availability

Metagenome sequencing data are available from the National Center for Biotechnology Information (NCBI) Sequence Read Archive (SRA) under accession number PRJNA916472, and the accession number of RNA-seq data is PRJNA917555.

## SUPPLEMENTAL MATERIAL

Supplemental figures msystems.01500-24-s0001.docx Figures S1 to S4.

Legends msystems.01500-24-s0002.docx Legends for supplemental files.

Table S1 msystems.01500-24-s0003.xlsx Composition and nutrient levels of experimental diets.

Table S2 msystems.01500-24-s0004.xlsx Primer sequences.

Table S3 msystems.01500-24-s0005.xlsx Comparison of 181 species (candidate biomarkers).

Table S4 msystems.01500-24-s0006.xlsx The names of 181 candidate species-level microbes corresponding to the microbial numbers.

Table S5 msystems.01500-24-s0007.xlsx The 52 upregulated genes in LW-10.5.

Table S6 msystems.01500-24-s0008.xlsx The 114 downregulated genes in LW-10.5.

ASM does not own the copyrights to Supplemental Material that may be linked to, or accessed through, an article. The authors have granted ASM a non-exclusive, world-wide license to publish the Supplemental Material files. Please contact the corresponding author directly for reuse.
