# Supplementary figures and images for: Interactions between gut microbes and host promote degradation of various fiber components in Meishan pigs
Source: mSystems. 2025 Jan 28;10(2):e01500-24. doi: 10.1128/msystems.01500-24 (PMC11834408; doi:10.1128/msystems.01500-24)

**Figure S1**


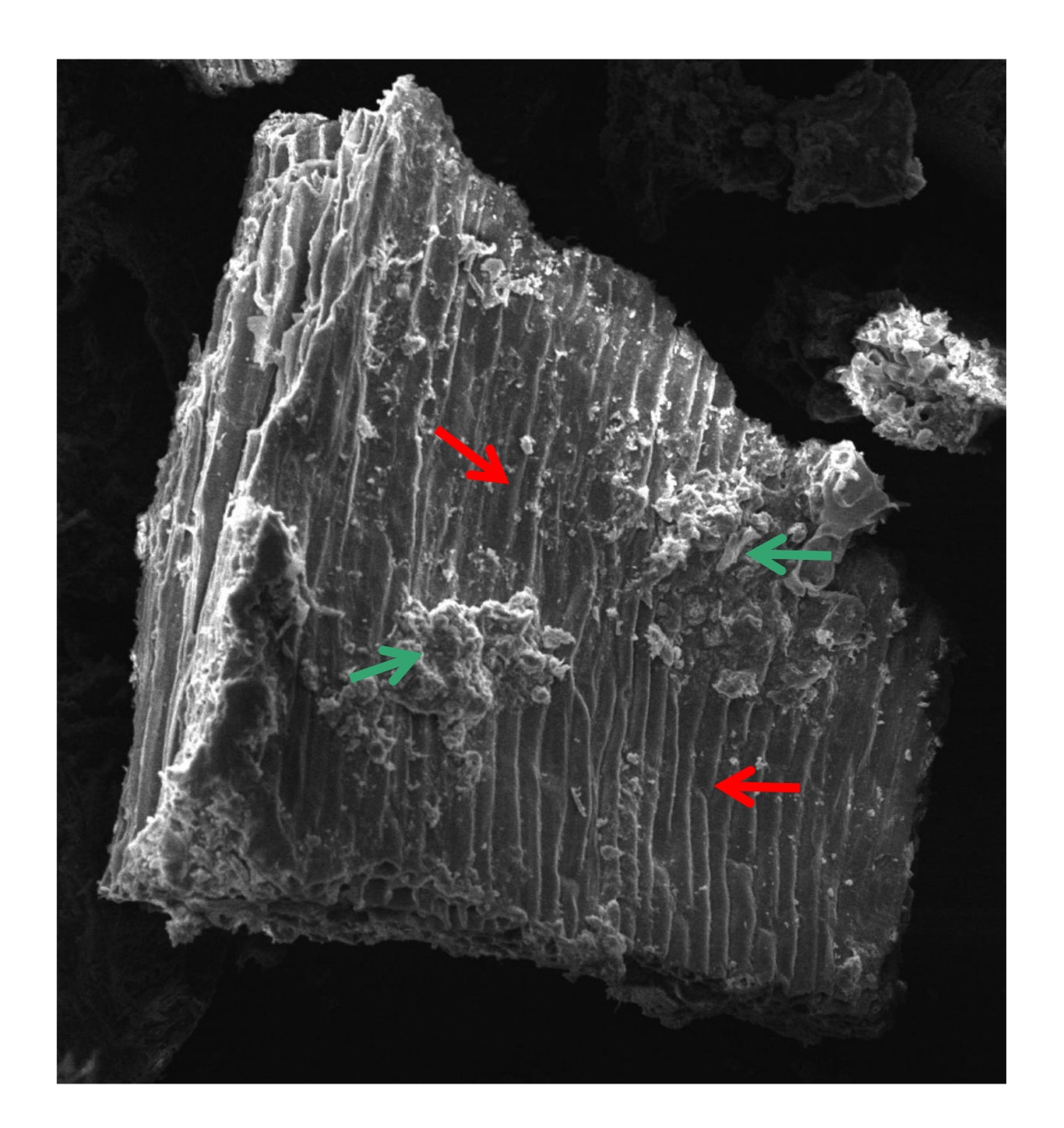


**Figure S2**





**Figure S3**





**Figure S4**

Supplement: Supplemental figures — Figures S1 to S4. [file msystems.01500-24-s0001.docx]
