# Supplementary material for: Interactions between gut microbes and host promote degradation of various fiber components in Meishan pigs
Source: mSystems. 2025 Jan 28;10(2):e01500-24. doi: 10.1128/msystems.01500-24 (PMC11834408; doi:10.1128/msystems.01500-24)
Supplement: Legends — for supplemental files. [file msystems.01500-24-s0002.docx]

**Supplemental tables**

**Table S1.** Composition and nutrient levels of experimental diets (DM basis %)

**Table S2.** The primer sequences of differentially expressed genes related to short-chain fatty acid transport in cecum for qRT-PCR.

**Table S3.** Comparison of 181 species (candidate biomarkers) between the control group and the WB-10.5 group

**Table S4.** The names of 181 candidate species-level microbes corresponding to the microbial numbers

**Table S5.** The 52 upregulated genes in LW-10.5

**Table S6.** The 114 downregulated genes in LW-10.5

**Supplemental figures**

**Figure S1** A scanning electron micrograph of undigested fodder-based plant tissue in feces. Red arrows indicate cellulose microfibrils and green arrows indicate matrix polysaccharide.

**Figure S2.** Annotative analysis of carbohydrate active enzymes in cecal microbiota. **A** The proportion of CAZymes at class level. **B** The main GH families (top 20) in each sample. **C** Major species possessing GH-encoding genes in SGS. MS, Meishan pigs. LW, Large White pigs. SGS, specific gene set.

**Figure S3.** Measurement of thickness of different layers of cecal intestinal wall based on HE staining. MS, Meishan pigs. LW, Large White pigs. WB, Wheat bran. * means *P*<0.05, ** means *P*<0.01.

**Figure S4.** Functional enrichment analysis of genes and quantitative validation of RNA seq accuracy. **A** Enrichment analysis of 166 genes. **B** In each pig breed, the RNA-seq of *GPR174*, *GPR183* and *SLG2A4* in WB-10.5, compared to the CON. **C** In each pig breed, the RT-PCR of *GPR174*, *GPR183* and *SLG2A4* of WB-10.5, compared to the CON. MS, Meishan pigs. LW, Large White pigs. The values are presented as log_2_ (fold change). **P* < 0.05, ** *P* < 0.01, *** *P* < 0.001
